# Validation of the Management of Emotions in Others Scale-Short Form in Spanish adolescents

**DOI:** 10.3389/fpsyg.2025.1677479

**Published:** 2026-01-28

**Authors:** Víctor Martín-Laguna, Alba Rodríguez-Donaire, Pablo Luna, Lidia Losada, Javier Cejudo

**Affiliations:** 1 Departamento de Psicología, Universidad de Castilla-La Mancha Facultad de Educacion, Ciudad Real, Spain; 2 Métodos de Investigación y Diagnóstico en Educación II, Universidad Nacional de Educacion a Distancia Facultad de Educacion, Madrid, Spain

**Keywords:** adolescence, factor structure, interpersonal emotion regulation, MEOs, reliability, validity

## Abstract

The Managing the Emotions of Others Scale—Spanish Adaptation Short Form (MEOS-SASF) is an instrument used to assess prosocial and non-prosocial aspects of interpersonal emotion regulation. The psychometric properties of the Spanish version of the scale have not been evaluated in adolescent populations. The aims of the present study were, first, to examine the factorial and convergent validity, internal consistency, and gender and age related measurement invariance of the Spanish adolescent adaptation of the MEOS-SF (MEOS-SASF), and second, to evaluate its applicability in an adolescent sample of 701 Spanish adolescents (*M* = 13.26; SD = 1.10). The MEOS-SASF presented a three-factor (prosocial, non-prosocial and concealing) correlated structure. The three subscales of the MEOS-SASF exhibited adequate internal consistency indices (alphas and omegas = 0.72–0.87). The results of the gender and age analysis revealed a good fit. Additionally, the MEOS-SASF showed evidence of convergent validity with respect to trait emotional intelligence, sadism, social competence and satisfaction with life. The MEOS-SASF offers reliable and valid measures of interpersonal emotion regulation in Spanish adolescents.

## 1 Introduction

Emotion management or regulation is a key domain of EI (Austin and O’Donnell, 2013), being included as a dimension in all its main theoretical models (Mayer and Salovey, 1997; Mayer et al., 2016; Petrides and Furnham, 2001; Petrides et al., 2016). Although EI and emotion regulation exhibit some theoretical overlap, they are regarded as distinct fields of research (Austin and O’Donnell, 2013; Petrides et al., 2011; Zaki and Williams, 2013). Nonetheless, some authors have sought to integrate these two areas of study (Hughes and Evans, 2018; Peña-Sarrionandia et al., 2015). The construct of emotion management or regulation includes individual emotional regulation, which consists of processes focused on one’s own emotions in order to achieve personal wellbeing, and interpersonal emotion management, referred to the formation and pursuit of goals designed to change one’s own or others’ emotions through social interactions (Zaki, 2020).

Interpersonal emotion management comprises two elements (Austin and O’Donnell, 2013): the prosocial facet—helping someone to manage their anxiety, for example—and the non-prosocial facet, which refers to influencing the feelings and behaviors of others for the benefits on one’s own interests. This latter facet has come to be known as “*emotional manipulation”* or the dark side of EI (Austin and O’Donnell, 2013; Austin et al., 2018; Petrides et al., 2011). Instruments designed to assess trait EI, such as the Trait Emotional Intelligence Questionnaire (TEIQue) (Petrides et al., 2007) or the Bar-On Emotional Quotient Inventory (EQ-i) (Bar-On, 1997) include sub-scales with items that refer to interpersonal emotion management. Although few scales include both prosocial and non-prosocial aspects, the Emotion Regulation of Others and Self Scale (EROS) (Niven et al., 2011) assesses emotion regulation strategies that seek to both improve (prosocial facet) and worsen (non-prosocial facet) the affect of others.

Given the need for reliable measures of interpersonal emotion regulation in youth, the present study focuses on validating a scale specifically applied to adolescents. Emotion regulation plays a crucial role in adolescence (Martínez-Yarza et al., 2023; Ruíz-Aranda et al., 2012; Taylor et al., 2017). Traditionally, developmental research has focused on the intrapersonal regulation of one’s own emotions, which progressively shifts from caregiver-supported regulation in early childhood toward greater autonomous self-regulation during adolescence. However, the management of others’ emotions constitutes a distinct process that belongs primarily to the domain of social development rather than to personal emotional regulation. Adolescents become increasingly embedded in complex peer contexts where empathic responding, prosocial behavior, and the ability to influence others’ emotional states become central to social functioning and interpersonal adjustment (Sahi et al., 2023). Despite this distinction, most validated Spanish-language measures for youth focus on intrapersonal emotion regulation. These include the Emotion Regulation Questionnaire (ERQ) for children and adolescents (Martín-Albo et al., 2020), that includes 10 items for the assessment of cognitive reappraisal and expressive suppression and the Cognitive Emotion Regulation Questionnaire (CERQ) (Betegón et al., 2022; Chamizo-Nieto et al., 2020), to evaluate cognitive emotion regulation strategies, both grounded on a cognitive perspective on emotion regulation. From a functional perspective, the Spanish Children’s Sadness Management Scale CSMS-E (Restrepo et al., 2021) to evaluate Coping, Inhibition and Dysregulation of sadness in adolescents. Finally, the Difficulties in Emotion Regulation Scale (DERS) (Gómez-Simón et al., 2014), approaches emotion regulation from a psychopathological framework, capturing deficits across multiple maladaptive dimensions. However, these instruments exclusively address intrapersonal aspects, without including non-prosocial dimensions. Furthermore, no versions of the MEOS, MEOS-SF or MEOS-VSF for adolescents have been identified in any language, despite the need for studies on age-related differences in interpersonal emotion management (Austin et al., 2018) and for research that clarifies the processes involved in the acquisition and development of mechanisms to regulate emotions during adolescence (Theurel and Gentaz, 2018).

Austin and O’Donnell (2013) developed the Managing the Emotions of Others Scale (MEOS), which comprises 58 self-report items based on the trait EI model for use with adult population. The purpose of the scale is to assess pro-social and non-prosocial aspects of interpersonal emotion management. It is composed of four core factors, two prosocial and two non-prosocial: *Enhance*, offering help or comfort to improve another’s mood; *Divert*, use of diversion to enhance another’s mood; *Worsen*, the use of criticism to undermine another’s confidence; *Inauthentic*, the use of inauthentic strategies such as flattery, sulking or false “niceness”; and two secondary factors: *Conceal*, concealing emotions from others; and *Poor emotion skills*, understood as the low self-perceived ability to influence the emotions of others. It is worth highlighting that the cross-pair correlations between the members of the enhance/divert and worsen/inauthentic subscales are comparatively weak, suggesting that individuals may use both prosocial and non-prosocial strategies to manage the mood of others (Austin and O’Donnell, 2013). The authors’ results revealed positive relationships between the non-prosocial factors and the Dark Triad traits of personality, while prosocial factors were negatively associated with the Dark Triad traits. Additionally, the results pointed to positive associations between the prosocial factors and trait EI, while the non-prosocial factors were negatively associated (Austin and O’Donnell, 2013).

To allow for the use of shorter scales and to facilitate their application in research settings where administering the full scale was not feasible, two brief versions were developed (Austin et al., 2018): the MEOS-SF (short form, with 30 items) and the MEOS-VSF (very short from, with 24 items) both validated with young adults. Both scales have a five-factor structure, with adequate evidence of internal consistency and validity; the poor skills subscale was omitted due to its unsatisfactory psychometric properties. The authors’ results confirmed that trait EI was positively associated with the prosocial factors and negatively associated with the non-prosocial factors (Austin et al., 2018). Finally, with regard to the relationships with subjective wellbeing, positive associations were found between the prosocial factors and positive affect in both versions, and negative associations were encountered between the non-prosocial factors and negative affect, while Enhance and Divert also showed weak positive associations with life satisfaction. These results are consistent with those reported in other studies (Austin and Vahle, 2016; Niven et al., 2011).

The MEOS has been adapted to Polish (Jankowski et al., 2016), maintaining the six-factor structure. It showed adequate internal consistency, with similar results to those of the original scale (Austin and O’Donnell, 2013). The authors delved into the relationship with ability-based EI, reporting an association between the prosocial factors and assimilation and emotion management and a practically null relationship between the inauthentic factor and ability EI (Jankowski et al., 2016). As in the study by Austin and O’Donnell (2013), positive associations were found between the non-prosocial factors and Dark Triad traits.

Two versions have also been developed in Mandarin. In the first version proposed, Saklofske et al. (2016) used the MEOS in its full form. Their factor analysis revealed a four-factor structure, with a merged Enhance/Divert factor and the omission of the Poor Skills factor, due to its poor psychometric properties. This new *Enhance/Divert* was positively associated with trait EI, with this association being more significant than in previous studies. The second version was adapted from the MEOS-SF, with the factor analysis supporting a three-factor model, which resulted from the merging of Enhance and *Divert* (prosocial factor) and *Worsen* and *Inauthentic* (non-prosocial factor), together with the *Conceal* dimension (Wang et al., 2021). Trait EI was found to be positively associated with the prosocial factor and negatively with the non-prosocial factor. The effect sizes were medium, which, according to the authors, suggests that individuals with high trait EI tend to use prosocial strategies while those with low trait EI tend to use non-prosocial ones (Wang et al., 2021). Additionally, the non-prosocial factor was found to be positively related to the Dark Triad traits while the prosocial factor was negatively associated. However, these versions have been validated with young adults and none of them has been designed for adolescent populations.

The need to adapt and validate an instrument assessing the management of others’ emotions specifically for adolescents arises from several empirical and theoretical considerations. Adolescence is a period characterized by profound social reorientation, during which interactions with peers become increasingly salient and interpersonal dynamics intensify (Crone et al., 2022; Ryan et al., 2019). In this context, adolescents frequently engage in behaviors aimed at influencing the emotions of others—both in prosocial ways, such as offering comfort or support, and in non-prosocial ways, such as teasing or manipulating peers. Despite the developmental relevance of these processes, no existing instruments assess prosocial and non-prosocial aspects of managing others’ emotions in adolescent populations in any language. A validated tool would also advance research on how interpersonal emotion management relates to key developmental outcomes, including social competence, wellbeing, and trait emotional intelligence or disadaptative behaviors (Bacon and Regan, 2016; Llamas-Díaz et al., 2022). Furthermore, this instrument could be readily incorporated into emotional education programs or longitudinal studies employing repeated-measures designs, thereby facilitating the evaluation of changes in interpersonal emotion regulation across adolescence (Zimmermann and Iwanski, 2014; McRae and Gross, 2020).

In short, the aims of our study are: (1) to provide evidence of the factorial validity of the Managing the Emotions of Others Scale—Spanish Adaptation Short Form (MEOS-SASF); (2) to demonstrate the internal consistency of the MEOS-SASF; (3) to analyze the invariance of the instrument in terms of sex, as studies have reported differences in the use of specific emotion regulation strategies according to sex (Johnson and Whisman, 2013) and age (Austin et al., 2018; Cracco et al., 2017; Theurel and Gentaz, 2018; Zimmermann and Iwanski, 2014); (4) to provide evidence of the convergent validity of the MEOS-SASF with respect to trait EI, sadism, social competence and life satisfaction; and (5) evaluate its applicability in an adolescent sample.

## 2 Materials and methods

### 2.1 Participants

The study sample comprised 701 adolescents aged between 12 and 16 years (early and middle adolescence), with a mean age of 13.26 years (SD = 1.10). Regarding age distribution, 30.4% (*n* = 213) were 12 years old, 30.8% (*n* = 216) were 13 years old, 24.0% (*n* = 168) were 14 years old, 11.8% (*n* = 83) were 15 years old, and 3.0% (*n* = 21) were 16 years old. The sample was balanced with respect to sex (49.9% girls, 50.1% boys).

All participants completed the Management of the Emotions of Others Scale—Short Form (MEOS-SF) (Austin et al., 2018) and the Adolescent Multidimensional Social Competence Questionnaire (AMSC-Q) (Gómez-Ortiz et al., 2017) (*n* = 701). A subsample completed the Trait Emotional Intelligence Questionnaire—Adolescent Short Form (TEIQue-ASF) (Petrides et al., 2006) and the Satisfaction with Life Scale (SWLS) (Diener et al., 1985) (*n* = 471). A smaller subsample completed the Assessment of Sadistic Personality (ASP) (Plouffe et al., 2017) (*n* = 286).

A non-probabilistic purposive sampling strategy was employed to recruit participants enrolled in compulsory secondary education. Inclusion criteria were: (a) being between 12 and 16 years of age and (b) being enrolled in an educational institution at the time of the study. Exclusion criteria comprised: (a) failure to obtain written informed consent from parents or legal guardians and (b) incomplete or invalid questionnaire responses.

An a priori statistical power analysis was conducted using G*Power 3.1 software (Faul et al., 2007), assuming a medium effect size (*f*^2^ = 0.15) and a desired statistical power of 90%. The results indicated a minimum required sample size of 358 participants. In the present study, this recommendation was exceeded. Moreover, several authors have recommended minimum ratios of between 5:1 and 10:1 participants per item to ensure a stable factor solution (e.g., Nunnally and Bernstein, 1994). Likewise, other authors suggest that an absolute sample size of at least 200 cases constitutes an adequate threshold for obtaining robust factor estimates (e.g., Kline, 2016).

### 2.2 Instruments

Management of Emotions in Others Scale—Short Form (MEOS-SF) (Austin et al., 2018). This scale assesses interpersonal emotion management. It comprises 30 items scored on a 5-point Likert-type scale (where 1 is strongly disagree and 5 is strongly agree). The MEOS-SF has five factors, mood-enhancing (Enhance), enhancing another’s low mood by diversion (Divert), mood worsening (Worsen), use of inauthentic strategies for self-serving intentions (Inauthentic), and concealing emotions from others (Conceal). The MEOS-SF was taken as the reference for the translation and validation of the scale in Spanish for an adolescent population.

Trait Emotional Intelligence Questionnaire—Adolescent Short Form (TEIQue-ASF) (Petrides et al., 2006), in its Spanish version by Ferrando et al. (2011). This scale measures trait emotional intelligence. It consists of 30 items that are scored on a 7-point Likert-type scale (where 1 is strongly disagree and 7 is strongly agree). Example items include “I can control my anger when I want to,” “I feel good about myself,” and “I’m good at getting along with my classmates.” In the present study, the reliability of this instrument, as measured by Cronbach’s alpha, was α = 0.83.

Assessment of Sadistic Personality (ASP) (Plouffe et al., 2017). This 9-item scale (based on the original 20-item version) measures everyday sadism. The scale was adapted and validated in Spanish for adolescents by Pineda et al. (2023). Participants rate the items on a Likert-type scale (0 = strongly disagree, to 4 = strongly agree). Example items include “I have made fun of people so that they know I am in control,” “I get pleasure from mocking people in front of their friends,” and “I think about hurting people who irritate me.” The reliability in our study was α = 0.78.

Adolescent Multidimensional Social Competence Questionnaire (AMSC-Q). This tool assesses social competence and has been validated in Spanish for adolescents (Gómez-Ortiz et al., 2017). The scale comprises 26 items that are scored on a 7-point Likert-type scale (1 = completely false; 7 = completely true). It addresses five domains of social competence: cognitive reappraisal, social adjustment, prosocial behavior, perceived social efficacy and normative adjustment. The present study used only the overall social competence score. Example items include “My classmates and friends come to me when they have a problem,” “My classmates feel comfortable working with me,” and “If a classmate is really overwhelmed and doesn’t have time to finish his/her work, I lend a helping hand.” Reliability in our study was α = 0.91.

Satisfaction with Life Scale (SWLS) (Diener et al., 1985), in its Spanish version for adolescents by Atienza et al. (2000). The SWLS is a short 5-item self-report questionnaire where people judge whether their life is satisfying on a 7-point rating scale, from 1 = strongly disagree to 7 = strongly agree. The scale provides a global measure of life satisfaction. It is related to the cognitive component of wellbeing. Example items include “My living conditions are excellent,” “I am satisfied with my life,” and “So far, I have achieved the important things I want in life.” Reliability in the present study was α = 0.81.

### 2.3 Procedure

First, for the process of validating the MEOS-SASF, we followed established guidelines for adapting instruments (Zenisky and Hambleton, 2012). The MEOS-SF (Austin et al., 2018) was translated into Spanish by a native English speaker with proficiency in Spanish. An independent translator subsequently back-translated the scale. Finally, the original and the translated items were analyzed and the authors reached a consensus on the final content of the instrument.

Second, the present research was approved by the Social Research Ethics Committee of the University of Castilla-La Mancha (Spain) (CEIS-646208-H2X8) framed within a national research project (PID2020-115624RA-I00) and partially supported by another (PID2023-151679OB-I00). Additionally, following the international ethical principles set out in the Declaration of Helsinki, appropriate measures were taken to ensure the complete confidentiality of the participants’ personal data, in accordance with Organic Law 3/2018, of December 5, on the Protection of Personal Data and Guarantee of Digital Rights.

Finally, once the content of the MEOS-SF had been developed and agreed upon, contact was made with the management team of the schools involved. The instruments were administered in digital format, by means of a questionnaire that the students completed on their mobile phones or tablets in the classroom. The confidentiality of the data was respected. To complete the questionnaires, informed consent was required from the families of students aged under 16 years.

### 2.4 Data analysis

For the analyses, first, descriptive statistics were performed to calculate the mean, standard deviation, skewness and kurtosis, as suggested by Ferrando et al. (2022) and Muñiz (2018). Second, we conducted a confirmatory factor analysis (CFA), for which the comparative fit indices (CFI) (Kline, 2016), Tucker-Lewis index (TLI), root mean square error of approximation (RMSEA) and standardized root mean square residual (SRMR) were used. The reference thresholds for the adjustment indices used are: CFI > 0.90, TLI > 0.90, RMSEA < 0.06–0.08, SRMR < 0.08. Third, we examined factorial invariance according to the sex and age group of the participants. Fourth, the internal consistency of the instrument was tested. Finally, the correlations between the MEOS-SAFS subscales and trait EI, sadism, social competence and life satisfaction were explored. All the analyses were conducted using the Rstudio program, with the Lavaan 0.6.18 and semTools 0.5.6 statistical packages.

## 3 Results

### 3.1 Descriptive statistics of the items

Table 1 shows the descriptive statistics of the items, with the mean ranging from 1.7 to 4.30 and the standard deviations from 0.84 to 1.30. With regard to the shape statistics, the skewness values range from -1.54 to 1.83 and those for kurtosis range from -1.03 to -3.13. These values are within those recommended for assuming univariate normal distribution (Curran et al., 1996; Kline, 2016).

**TABLE 1:** Descriptive statistics of the items.

Order	Items	*M*	SD	g_1_	g_2_
1.	If I want someone to do something for me, I am especially nice to them before asking.	3.55	1.11	-0.48	-0.37
2.	I sometimes use humour to try to lift another person’s mood.	4.25	0.90	-1.37	1.99
3.	I sometimes sulk to make someone feel guilty.	2.07	1.15	0.91	-0.02
4.	If someone’s behavior has caused me distress, I try to make them feel guilty about it.	2.61	1.19	0.33	-0.73
5.	When someone has made me upset or angry, I often conceal my feelings.	3.02	1.27	-0.05	-1.01
6.	If I want someone to do something for me, I try to elicit sympathy from them.	3.40	1.08	-0.35	-0.34
7.	When someone has made me upset or angry, I tend to downplay my feelings.	3.01	1.14	-0.04	-0.63
8.	I sometimes put someone down in public to make them feel bad.	1.57	0.99	1.83	2.78
9.	If I don’t like someone’s behavior, I make negative comments in order to make them feel bad.	2.02	1.10	0.96	0.20
10.	I use anger to get others to do things that I want them to do.	1.78	1.04	1.42	1.49
11.	When someone is unhappy, I try to cheer them by talking about something positive.	4.26	0.91	-1.54	2.65
12.	I don’t believe in telling others about my problems—I keep them to myself.	3.08	1.30	0.00	-1.03
13.	If someone tries to make me feel better when I am feeling low, I pretend to feel happier to please that person.	3.37	1.24	-0.41	-0.75
14.	If someone is anxious, I try to reassure them.	4.30	0.84	-1.54	3.13
15.	I use criticism to make others feel that they should work harder.	2.16	1.11	0.73	-0.16
16.	I often conceal feelings of anger and distress from others.	3.23	1.25	-0.24	-0.87
17.	When someone is unhappy, I show that I understand how they are feeling.	4.03	0.90	-1.04	1.32
18.	I know how to make someone feel ashamed about something that they have done in order to stop them from doing it again.	2.42	1.23	0.54	-0.64
19.	When someone is in a bad mood I try to divert them by telling jokes or funny stories.	3.91	1.04	-0.99	0.63
20.	When someone is under stress, I try to boost their confidence in their ability to cope.	3.90	0.98	-0.84	0.52
21.	I hide my feelings so others won’t worry about me.	3.36	1.26	-0.24	-0.98
22.	I sometimes use flattery to gain or keep someone’s good opinion.	2.56	1.14	0.29	-0.66
23.	When someone is anxious about a problem, I try to help them work out a solution.	4.20	0.89	-1.37	2.33
24.	If someone is feeling anxious, I try to calm them down by talking with them.	4.18	0.85	-1.21	2.05
25.	I sometimes sulk to get someone to change their behavior.	2.55	1.22	0.37	-0.75
26.	If someone is being awkward, I try to defuse the situation by being cheerful and pleasant.	3.70	1.00	-0.64	0.18
27.	If someone is angry, I try to divert their mood by being cheerful.	3.69	0.97	-0.61	0.26
28.	I can make someone feel anxious so that they will act in a particular way.	2.01	1.17	1.08	0.32
29.	When someone is in a bad mood I try to divert them by telling jokes or funny stories.	3.81	1.06	-0.85	0.32
30.	If someone has a problem I offer to help if they need it.	4.22	0.86	-1.18	1.68

g_1_, skewness; g_2_, kurtosis.

### 3.2 Evidence of factorial validity

**FIGURE 1:**
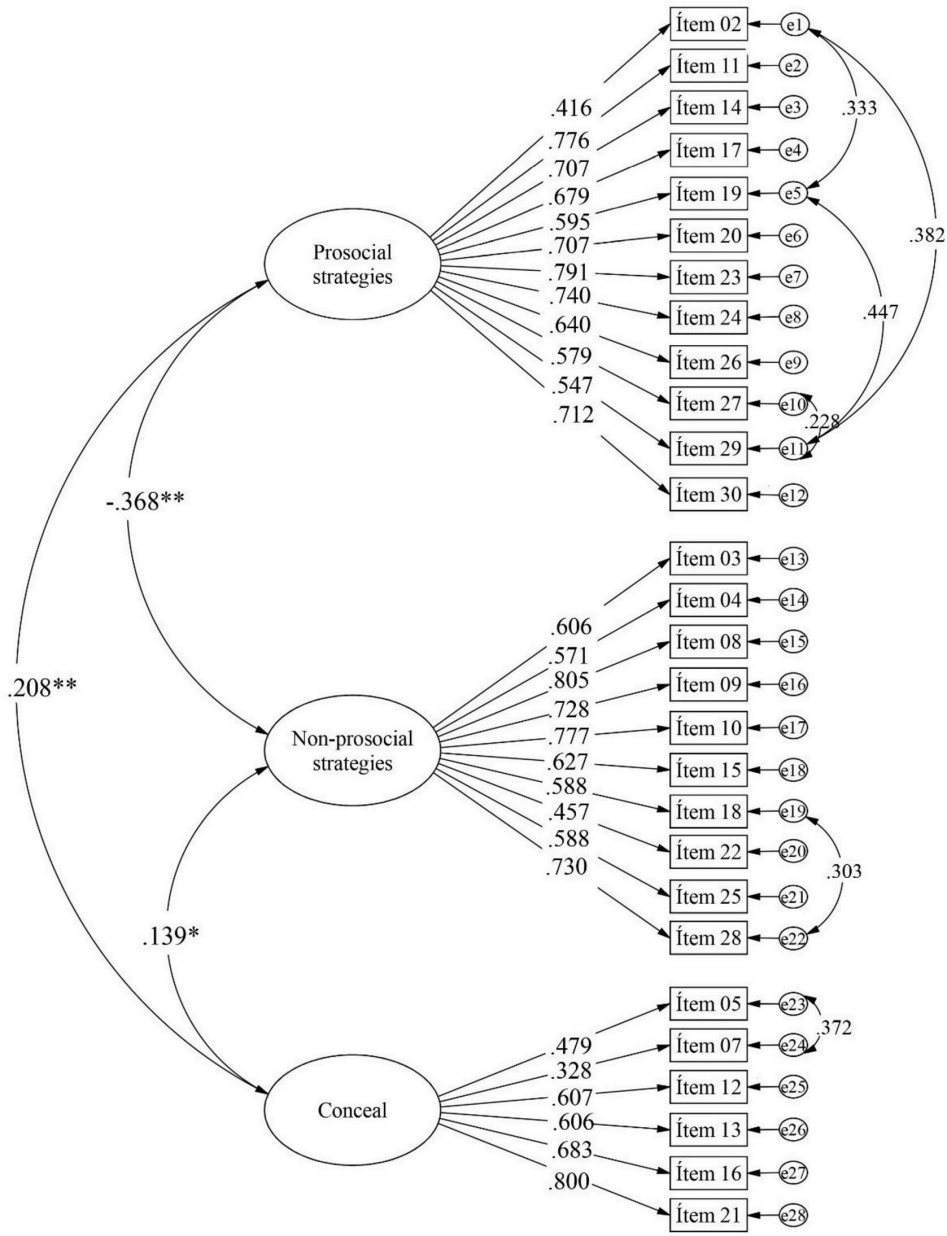
Factor loadings of the MEOS-SASF obtained from the CFA. The figure does not include Items 1 and 6. **p < 0.01, *p < 0.05.

For the CFA, we followed the recommendations of Lloret-Segura et al. (2014), whereby items should have at least five response options for them to be considered continuous, which is the case of the present instrument. Before the factor analysis, we examined whether the items met the criteria for multivariate normal distribution using Mardia’s test (kurtosis 89.96 with *p* < 0.05). As this criterion was not met, the Diagonally Weighted Least Squares (DWLS) estimator was used, as it can be applied for both linear and non-linear cases, currently being the most widely used and recommended method (Brown, 2015; Ferrando et al., 2022). In our first analysis, to examine the original five-factor model proposed by Austin et al. (2018) which included all the items, the indices for CFI and TLI were considered inadequate. In this model, Items 1 and 6 presented factor loadings below.30. Subsequently, the CFA led us to drop Items 1 and 6, resulting in acceptable fit indices. Table 2 shows these results. In Model 2, to examine the three-factor model proposed by Wang et al. (2021), the fit indices were considered acceptable. Additionally, the factor loadings for the three dimensions ranged from 0.328 to 0.805, as shown in Figure 1

**TABLE 2:** Fit indices for the two models in the CFA.

Models	*X* ^2^	*CFI*	*TLI*	*RMSEA*	*SRMR*
Model 1	1523.99**	0.897	0.887	0.064	0.075
Model 2	1082.15**	0.930	0.923	0.056	0.065

CFI, comparative fit index, TLI, Tucker-Lewis index, RMSEA, Root Mean Square Error of Approximation; SMRS, standardized root mean square residual. **p < 0.01.

As can be seen in Table 3, the prosocial strategy factor is negatively correlated, albeit weakly, with the non-prosocial strategy factor. Meanwhile, the conceal factor is positively, but weakly, correlated with both prosocial and non-prosocial strategies.

**TABLE 3:** Factor score bivariate correlations for the MEOS-SASF.

Factors	1	2	3
1. Prosocial strategies	–		
2. Non-prosocial strategies	-0.20**	–	
3. Concealing	0.21**	0.14**	–

**p < 0.01.

### 3.3 Internal consistency evidence

Table 4 presents the evidence for the internal consistency of the instrument. To provide internal consistency evidence, we calculated Cronbach’s Alpha coefficient and McDonlad’s omega (Dunn et al., 2014; Trizano-Hermosilla and Alvarado, 2016). Values over 0.70 were obtained, suggesting the instrument exhibits adequate internal consistency.

**TABLE 4:** Instrument reliability.

Factors	α	ω
1. Prosocial strategies	0.875	0.844
2. Non-prosocial strategies	0.843	0.836
3. Conceal	0.730	0.721

α, Cronbach’s alpha; ω, McDonald’s omega.

### 3.4 Measurement invariance

Finally, invariance was examined in order to verify the fairness of the participants’ MEOS-SASF scores, taking sex (male-female) and age group (12–13 years -14 to 16 years) into account. For invariance, the thresholds are: ΔCFI < 0.010 y ΔRMSEA < 0.015. The differences in CFI and RMSEA are within the accepted limits (Brown, 2015; Cheung and Rensvold, 2002), such that all the levels of measurement invariance (configurational, metric, scalar and strict) are met. This indicates that the instrument reliably measures an identical factor structure for the MEOS in both sex groups (male and female) and both age groups, ensuring that comparisons between these groups are legitimate and free of bias. The results are shown in Table 5.

**TABLE 5:** Invariance measures by gender and age group.

Models	X^2^	df	CFI	RMSEA	ACFI	ARMSEA
**Sex (female vs. male)**						
Configurational	1477.35**	682	0.914	0.058	–	–
Threshold	1545.98**	738	0.913	0.056	-0.001	-0.002
Metric	1534.93**	763	0.917	0.054	0.003	-0.004
Scalar	1602.45**	788	0.912	0.054	-0.005	0
Strict	1626.07**	816	0.913	0.053	0.001	-0.001
**Age group (early vs. middle)**						
Configurational	1495.92**	684	0.922	0.058	–	–
Threshold	1574.66**	740	0.919	0.057	-0.003	-0.001
Metric	1578.05**	765	0.921	0.055	0.002	-0.002
Scalar	1587.11**	790	0.923	0.054	0.002	-0.001
Strict	1608.31**	818	0.924	0.053	0.001	-0.001

This analysis accounts for the variable of participant gender, df, degrees of freedom; ΔCFI, variance in CFI; ΔRMSEA, variance in RMSEA. **p < 0.01.

### 3.5 Convergent validity evidence

For convergent validity (Table 6), the prosocial strategies subscale was positively correlated with trait emotional intelligence, social competence, and life satisfaction, and negatively correlated with sadism, while the non-prosocial strategies subscale was negatively correlated with trait EI, social competence, and life satisfaction, and positively correlated with sadism. The conceal subscale revealed negative correlations with trait emotional intelligence and life satisfaction.

**TABLE 6:** Correlations between the MEOS-ASF factors and trait EI, sadism, social competence and life satisfaction.

Factors	Trait EI *n* = 471	Sadism *n* = 286	Social competence *n* = 701	Life satisfaction *n* = 471
Prosocial strategies	0.24**	-0.18**	0.52**	0.24**
Non-prosocial strategies	-0.17**	0.62**	-0.25**	-0.03
Conceal	-0.39**	0.03	-0.05	-0.33**

**p < 0.01.

## 4 Discussion

The aim of our study was to provide evidence of the factorial validity and reliability of the subscales of the MEOS-SASF, a self-report instrument designed to assess the subjective perception of managing the emotions of others in Spanish adolescents. It also sought to analyze the invariance of the MEOS-SASF across sex and age. Finally, the study also aimed to examine the convergent validity of the MEOS-ASF in relation to trait EI, sadism, social competence and life satisfaction.

First, the results of the CFA provided evidence for a three-factor structure of the MEOS-SASF, comprising 28 items (two were omitted due to their low factor loadings): (1) Prosocial factor, comprising items related to the tendency to improve the emotional state of others, use of humor or distraction, (2) Non-prosocial factor, which includes behaviors aimed at intensifying negative emotions in others or the use of insincere or feigned strategies and (3) Conceal, which refers to behaviors that involve inhibition or suppression of emotional expression, whether for prosocial or non-prosocial purposes. First, the comparison of fit indices favored the final three-factor solution. Although both models achieved adequate levels of overall fit, the selected model showed slightly higher CFI/TLI values and lower RMSEA and SRMR values. Second, we prioritized model parsimony, a key psychometric principle according to which, between two solutions with comparable fit, the one requiring fewer parameters and exhibiting greater structural simplicity should be preferred. In this case, the final model adequately replicated the latent structure with fewer factors and reduced complexity, without compromising the interpretation of the construct. This is consistent with the findings in adults reported by Wang et al. (2021). The resulting factor structure was obtained by merging dimensions of the original MEOS (Austin and O’Donnell, 2013) and the MEOS-SF (Austin et al., 2018): Enhance and Divert (prosocial factor), Worsen and Inauthentic (non-prosocial factor) and Conceal (Wang et al., 2021). However, the six-factor structure proposed for the original MEOS (Austin and O’Donnell, 2013) and its Polish adaptation (Jankowski et al., 2016) was not replicated in our sample. Furthermore, our results also contradict the five-factor structure identified for the MEOS-SF and MEOS-VSF (Austin et al., 2018).

On the other hand, it is necessary to comment on the issue of residual correlations. In CFA, the presence of residual correlations indicates that certain items share variance not explained by the latent factors, which may reflect content similarity, wording effects, or specific subdimensions (Brown, 2015; Kline, 2016). Following the recommendations of Raykov and Marcoulides (2006), these correlations were examined as indicators of potential model refinements. In our case, the residuals cluster among groups of items with very similar content. Although their presence does not invalidate the overall factor structure, it does suggest areas where the instrument could be improved conceptually or through future wording adjustments.

To provide evidence of internal consistency, we calculated Cronbach’s alpha and McDonald’s omega coefficients. The results showed satisfactory internal reliabilities for the MEOS-SASF subscale scores. When compared to the results obtained by Wang et al. (2021), the reliability of the different subscales of the MEOS-SASF are slightly higher for the prosocial strategies factor, similar for the non-prosocial strategies factor, while, for the conceal factor, they are higher. The factor invariance analysis verified that this structure is maintained with respect to age and sex, enabling us to perform a joint analysis by age group and sex, as in previous studies (Austin et al., 2018; Austin and O’Donnell, 2013; Jankowski et al., 2016; Saklofske et al., 2016; Wang et al., 2021).

To provide proof of convergent validity, we examined the correlations with trait EI, sadism, social competence and life satisfaction. Trait EI was found to be positively associated with the prosocial strategies factor (*r* = 0.24, *p* < 0.01) and negatively with the non-prosocial strategies factor (*r* = -0.17, *p* < 0.01) and conceal (*r* = -0.39, *p* < 0.01). These results are consistent with the findings of other studies using different versions of the MEOS (Austin et al., 2018; Austin et al., 2014; Austin and O’Donnell, 2013; Jankowski et al., 2016; Wang et al., 2021). The correlations obtained in the present study are, however, weaker than those found in adult population, which might suggest that adolescents with both high and low IE may harness both types of emotion regulation strategies at the same time. Another possible explanation for these findings, following Austin and O’Donnell (2013), may lie in the heterogenous nature of the TEIQue-ASF scale, which comprises two items from each of the 15 subscales of the full TEIQue (Petrides and Furnham, 2006).

In addition, our analysis of the associations between the dimensions of the MEOS-SASF revealed that the prosocial strategies factor (Enhance/Divert) was significantly and negatively correlated with the non-prosocial strategies factor (Worsen/ Inauthentic), being consistent with findings of other studies using adult samples (Austin and O’Donnell, 2013; Austin et al., 2018; Saklofske et al., 2016). The size of the correlation was, however, small (*r* = -0.20, *p* < 0.01), coinciding with the results of previous research (Austin and O’Donnell, 2013; Saklofske et al., 2016; Wang et al. (2021). This arguably suggests that, during adolescence, the use of prosocial strategies only slightly reduces the use of non-prosocial strategies, rather than strongly inhibiting them, which aligns with the findings of other studies (Austin and O’Donnell, 2013; Austin et al., 2018; Jankowski et al., 2016; Saklofske et al., 2016, Wang et al., 2021). It is worth noting that this finding is consistent with those of Niven et al. (2011), who reported a moderate association between the extrinsic affect-improving and affect-worsening factors of the EROS scale (Niven et al., 2011). Meanwhile, we found the conceal factor to be positively, but weakly, related to the prosocial strategies factor (*r* = 0.21, *p* < 0.01) and the non-prosocial strategies factor (*r* = 0.14, *p* < 0.01). These data thus suggest that adolescents may use the conceal strategy, in some cases, to improve mood, and in others, to worsen it, which aligns with the findings of Wang et al. (2021). This factor captures behaviors aimed at hiding or modulating the visible expression of emotions in interpersonal contexts. This construct should be distinguished from intrapersonal expressive suppression, which refers to the inhibition of one’s emotional expression primarily for the regulation of internal affect. Although these processes are conceptually distinct, they may interact in adolescence: concealment can serve interpersonal goals (e.g., preserving relationships, avoiding conflict) or self-protective purposes (e.g., avoiding vulnerability or gaining social advantage). While context-dependent suppression or concealment may be adaptive under certain circumstances (Harrington et al., 2020; Lougheed and Hollenstein, 2012; Westphal et al., 2010), chronic use of response-focused inhibitory strategies could be associated with maladaptive patterns such as psychological rumination. Given that the Conceal factor involves response-focused regulation (Gross, 2015; McRae and Gross, 2020), future studies should examine the differential effects of concealment depending on its primary motivation (prosocial vs. self-serving), and explore its prospective relationship with rumination (Cann et al., 2011). This could be achieved by combining measures of cognitive emotion regulation (e.g., CERQ) (Chamizo-Nieto et al., 2020) with brief motive-based items specifically assessing adolescents’ intentions when concealing their emotions.

Sadism, on the other hand, was positively related to the non-prosocial strategies factor (*r* = 0.62, *p* < 0.01) and negatively to the prosocial one (*r* = -0.18, *p* < 0.01), while no association was found with the conceal factor. This is consistent with the tendency toward interpersonal manipulation that characterizes this dark personality trait, which is in line with the findings of other studies on the associations with the Dark Triad of personality in adults (Austin et al., 2018; Austin and O’Donnell, 2013; Jankowski et al., 2016; Saklofske et al., 2016; Wang et al., 2021). Social competence was positively related to the prosocial strategies factor (*r* = 0.52, *p* < 0.01) and negatively to the non-prosocial strategies factor (*r* = -0.25, *p* < 0.01), while no association with conceal was found. In this sense, these moderate associations suggest that adolescents with high social competence may tend to leverage prosocial emotional regulation strategies, while those with low social competence do the opposite. Life satisfaction was positively related to the prosocial strategies factor (*r* = 0.24, *p* < 0.01) and negatively with conceal (*r* = -0.33, *p* < 0.01). These associations coincide with the conclusions of other studies reporting direct relationships between the use of prosocial emotion regulation strategies and affective wellbeing (Austin et al., 2018; Niven et al., 2012).

The present study is not without limitations. First, we used non-randomized convenience sampling, while randomized sampling would be needed to facilitate the extrapolation of the results. Second, only self-report scales were used, while maximum performance instruments (e.g., ability emotional intelligence, intelligence) would have been useful). Third, we did not measure test-retest reliability, as was done in other studies on versions of the MEOS (e.g., Austin et al., 2018). Fourth, more extensive validation of the MEOS-SASF, using personality and Dark Triad scales adapted for adolescents, would be of interest. In addition, to determine the effect size, G*Power 3.1 software was used, although theoretically it would have been more appropriate to use SEmPower. However, the use of *G*Power was considered appropriate since it is based on expected effects derived from previous empirical literature. Notwithstanding these limitations, the present study is the first to provide empirical evidence of the validity and reliability of the MEOS-SASF in Spanish adolescents. As expected, the MEOS-SASF was found to be a useful, reliable and valid measure to assess interpersonal emotion management in adolescent population. Specifically, the MEOS-SASF shows reliability and validity among Spanish adolescents and demonstrates measurement invariance across sex and different age groups. Consequently, having access to a brief and adapted instrument to measure how adolescents use prosocial and non-prosocial emotion regulation strategies broadens our knowledge of emotion regulation, which may have a substantial influence on this population, while also contributing to the future exploration of interpersonal emotional manipulation in adolescent population.

## Data Availability

The datasets presented in this study can be found in online repositories. The names of the repository/repositories and accession number(s) can be found in this article/Supplementary material.
